# “Resources‐Demands Ratio”: Translating the JD‐R‐Model for company stakeholders

**DOI:** 10.1002/1348-9585.12101

**Published:** 2019-11-27

**Authors:** Gregor J. Jenny, Georg F. Bauer, Désirée Füllemann, Sylvia Broetje, Rebecca Brauchli

**Affiliations:** ^1^ Center of Salutogenesis Division of Public and Organizational Health, Epidemiology, Biostatistics and Prevention Institute University of Zurich Zürich Switzerland; ^2^ Applied Psychology University of Applied Sciences and Arts Northwestern Switzerland Olten Switzerland

**Keywords:** employee surveys, JD‐R model, organizational change, translational research

## Abstract

**Objectives:**

Practitioners and organizational leaders are calling for practical ways to explain and monitor factors that affect workplace health and productivity. This article builds on the well‐established Job Demands‐Resources (JD‐R) model and proposes an empirically tested ratio that aggregates indicators of job resources and demands. In this study, we calculate a ratio of generalizable job resources and demands derived from the JD‐R model and then translate the ratio into the language of company stakeholders.

**Methods:**

We calculated a ratio based on measures applied in a large stress management intervention study (n = 2983) and report the findings from cross‐sectional analysis with health and productivity outcomes from same‐source and separate‐source data.

**Results:**

Findings showed a strong and unambiguous increase in health and productivity measures with each step of increase in the ratio. Loss in explained variance due to aggregation of two factors into a single ratio is small for measures which are known to be predicted by both factors simultaneously.

**Conclusions:**

A translation and visualization of the ratio that is accessible to practitioners and organizational leaders is presented and its use in companies discussed.

## 1 INTRODUCTION

In the field of research on work, stress and health, three models have gained recognition that explain the relationship between working conditions on the one side and health deterioration or motivational improvements on the other. Siegrist's Effort‐Reward‐Imbalance (ERI) model^1^ shows how high efforts combined with low rewards can lead to negative health outcomes. Karasek's Demand‐Control (DC) model^2^ illustrates how high demands at work combined with low control will strain employees. Bakker and Demerouti's Job Demands‐Resources model (JD‐R)^3^, ^4^ includes a wider range of job resources and demands, describing two distinct processes: a positive, motivational process and a negative, health‐impairing process. All three models underline that a combination of aggregated or selected job demands and resources leads to both desirable and undesirable outcomes. For the ERI model as well as the DC model, ratio measures have been developed that illustrate at a glance the balance between efforts and rewards or between demands and control, respectively. Regarding the ERI model, the use of a dichotomous variable is prevailing, indicating simply whether it is efforts or rewards that dominate. Some research though, has also used a logarithmically transformed, continuous ERI ratio.^5^ The DC model is researched most commonly with high and low strain groups, but also with separate predictors and interaction terms.^6^

### 1.1 Condensation of information for company stakeholders

All these approaches imply a condensation of information, which has high potential for both practical use and for intervention and evaluation research in the field of work, stress and health, utilizing global outcome indicators for project reporting. Condensing information into simple indices based on models explaining the links between job characteristics and health outcomes is usually not of particular interest to researchers, because condensation, ie the aggregation and transformation of continuous scales into ratios, difference scores, ordinal or even nominal groups, is usually accompanied by a loss in variance. However, there is a call from consultants, managers and experts in companies dealing with work and health issues asking for more accessible ways to present information to professionals at different hierarchical and functional levels in companies. This could be achieved by presenting information at several degrees of complexity, for example a condensed 'index' to report to top management; a comprehensible and manageable set of factors for middle management and employees; and a detailed set of scales for experts. Experts are usually executives from areas such as occupational health and safety management (OHS), worksite health promotion and human resources management (HRM) who need to plan, evaluate and report on the issue. Mostly, these experts are interested in scientifically valid scales and information, but with limits to length and complexity and a need for aggregation. The extent to which indices based on the ERI, DC or JD‐R model have found their way into the practice of consulting and management is unknown. For the JD‐R model, this practical use has explicitly been put forward by Schaufeli and Taris, who believe that the model could bridge occupational health management and human resources management by addressing both health and performance outcomes.^4^ In a similar line, we suggest that by condensing information on the 'input' side—ie combining job resources and demands into a ratio—the practical use of this highly popular model could be further enhanced. While the ERI and DC models already offer a way of condensation but are limited to specific job demands and resources, we suggest calculating a ratio of generalizable job resources and demands derived from the JD‐R model.

### 1.2 Considerations when computing a ratio and formulation of hypotheses

The following questions need to be considered when computing a ratio of job resources and demands based on the JD‐R model: (a) What kind of job resources and demands have to be selected to compute a ratio? (b) What mechanisms of interaction between job resources and demands are postulated and how do these affect the predictive power of the ratio? (c) What are the benefits of communicating a ratio over single factors? *Ad* (1) The practical use of a ratio for company stakeholders and consultants depends on its generalizability (for other approaches see for example Nielsen et al.^7^). Such indicators need to be comparable within and also across companies, ideally with benchmark data—otherwise potentially idiosyncratic information is presented to stakeholders and agents of change with unclear relevance outside the company, possibly providing little impetus for action. For this reason, the ratio calculated in this study is based on a general selection of job resources and demands applied in a large stress management intervention study: The JD‐R model computed with these scales proved to be invariant across companies (industry, services, health care, public administration), gender, leadership function and time—which means that both the motivational and health‐impairing regression paths based on these scales are of relevance for a broad spectrum of companies and employees.^8^, ^9^ *Ad* (2) The JD‐R model describes two distinct processes, but also their interaction. Especially the buffering impact of job resources on the health impairment process has been subject of much research, and Schaufeli and Taris state that “(...) in order to understand one process, the other process should also be taken into account, and vice versa (...)” (p. 57).^4^ Depending on the path and the chosen outcome variable of the JD‐R model, the model appears to be stronger at predicting burnout (which is affected by both job demands and resources) than work engagement (which is predominantly affected by job resources). Furthermore, some studies have shown that there are conditional effects, ie when job demands or challenges are high, then the impact of job resources on work engagement is accentuated.^10^ This is similar to the DC model that postulates active jobs when both demands and control are high, although corresponding evidence is limited.^11^ Evidence on conditional effects in the JD‐R model seems to depend on the operationalization of resources, and especially on that of demands.^10^ For this reason, Schaufeli and Taris define job demands as “(…) *negatively valued* physical, mental, social or organizational job characteristics that require sustained physical or psychological effort and are associated with physiological and/or psychological costs” (p. 56), which excludes so‐called challenging demands.^4^ The scales used in this study are mainly operationalized in this way, for example, work is “too difficult” or one “cannot work on something in peace” (see methods section). Such discussions also revolve around the definition of (job) resources and their possibly differential effect depending on context,^12^ and value‐based solutions have been proposed—see again Schaufeli and Taris, who define job resources as “(…) *positively valued* physical, mental, social or organizational job characteristics that are functional in meeting job requirements, reduce the associated physiological and/or psychological costs and stimulate personal growth and development” (p. 56).^4^ The scales used in this study cover social and task‐related resources that most broadly cover these functions (eg, support, appreciation, control). Nevertheless, when applying a ratio it must be clear that it is the starting point of a differentiated discussion and not its end point (see conclusions and limitation). Finally, research shows that a ratio or quadrant approach to ERI, DC or JD‐R is not superior in explaining variance in outcomes compared to two independent predictors.^13^, ^14^, ^15^, ^16^ As reported, when using resources and demands as independent variables, their influence on relevant organizational outcomes has been well established in previous research. We expect that the calculation of a ratio of resources to demands will follow this same pattern, while being accompanied by a small although not significant loss in variance. Based on this reasoning, we formulate the following two research hypotheses:

Hypothesis 1 An increase in the ratio is significantly associated with health and productivity outcomes in terms of decreased disorders and increased motivation and well‐being.

Hypothesis 2 The variance explained in health and productivity outcomes by the ratio is comparable to the variance explained by the single factors.

*Ad* (3) The decision to condense information into quadrants or ratios is mainly one of communication. The ratio approach has the natural appeal of a balance between job demands and resources—ie a state of harmony and equality in danger of disturbance through change in demands and the need for an increase in resources to regain balance. We deliberately computed a ratio of job resources with job demands as the denominator and not vice versa, stressing the importance and build‐up of job resources for counterweighing job demands and for their motivational potential resulting in work engagement and productivity—comparable to salutogenic^8^ and positive psychological approaches such as the positivity ratio by Fredrickson.^17^ While these ratios can be used as continuous scales, communication may be eased by categories with defined ranges of the ratio as markers. There are several ways to apply such markers—through means and standard deviations, benchmarks, or percentiles—which generate group affiliations and valuations. Such groups then serve as baseline and target of change processes, for example, an intervention project could aim to double the percentage of employees with a good to very good ratio. Finally, a label needs to be applied to this ratio which appeals both to stakeholders focused on occupational health and human resource management.

## 2 MATERIALS AND METHODS

### 2.1 Sample & design

The present study used data collected in the context of a large‐scale stress management intervention program (see acknowledgements). This program was implemented in eight medium and large Swiss organizations in diverse sectors (industrial, service, public administration, health care) and different regions (French and German‐speaking) from 2008 to 2011. The baseline online survey in 2008 yielded a sample of 2983 participants (response rate: 71%). The average age was 38.7 (SD = 11.3) and 29.1% of respondents reported having a supervisory role. Women represented 40.7% of the sample. Participants completed the newly developed, multi‐language online survey JSA (Job Stress Analysis) comprising a basic section with 35 validated scales on stressors, resources, well‐being and health. While the participants completed the questionnaire at one time point and are labeled “same‐source” data in the analysis, a subsample of the participants also completed an additional health questionnaire at another time point and are labeled “separate‐source” data. This health questionnaire covered a list of disorders, visits to medical services, consumption of drugs and insurance coverage and was returned by 964 employees, 550 of whom could be matched with the baseline survey data. No ethical review was necessary under national, university or departmental rules. The study was conducted under strict observation of ethical and professional guidelines.

### 2.2 Measures

All participants completed the base questionnaire that assessed self‐rated job demands, job resources, and health and productivity outcomes as well as basic demographic questions. A subset of the sample also completed the additional health questionnaire. The basic set of scales had first been selected by stress researchers in Switzerland, based on two existing instruments for ‘stress‐related job analysis’ (ISTA)^18^ and ‘salutogenic subjective work analysis’ (SALSA),^19^ covering stressors and resources known from research literature being key for well‐being and health. This selection of scales was made together with stress management consultants, to make sure they were applicable to the heterogenous companies (see acknowledgements). Second, a stepwise qualitative and quantitative procedure of scale selection and structural equation modeling yielded a JD‐R model with the following study scales, invariant across companies, gender, leadership function and time, as mentioned above.^8^, ^9^

#### 2.2.1 Job demands

*Time pressure* and *work interruption* were assessed with four items (*α* = .83) ranging from 1 = *very rarely/never* to 5 = *very often/constantly* (eg 'At work, how often is a rapid pace of work required?' and 'How often does it occur that you cannot work on something in peace because something else always comes in between?').^18^ *Qualitative overload* was assessed with three items (*α* = .83) ranging from 1 = *almost never/not at all true* to 5 = *almost always/fully true* (eg 'It happens that the work is too difficult for me'.).^19^ *Uncertainty at work* was assessed with four items (*α* = .75) ranging from 1 = *very rarely/never* to 5 = *very often/constantly,* and with one item ranging from 1 = *from nobody* to 5 = *from more than three persons* (eg 'How often do you receive ambiguous instructions?').^18^

#### 2.2.2 Job resources

*Supportive leadership* was assessed with five items (*α* = .82) ranging from 1 = *almost never/not at all true* to 5 = *almost always/fully true* (eg 'The line manager lets one know how well a job has been done'.).^19^ *Interpersonal fairness* was assessed with four items (*α* = .81) ranging from 1 = *to a small extent* to 5 = *to a large extent* (eg 'He/she treated you with respect?').^20^ *Manager* and *peer support* were assessed with one item each ranging from 1 = *not true at all* to 5 = *a lot* ('How much can you rely on the following people in difficult situations at work?').^21^ *Manager* and *peer appreciation* were assessed with one item each on a 7‐point graphical scale using *smileys* (eg 'Overall, how satisfied are you with the appreciation of your person shown by your line manager?').^22^ *Holistic tasks* were assessed with one item ranging from 1 = *almost never/not at all true* to 5 = *almost always/fully true* (eg 'In my job one can produce something or carry out an assignment from A to Z'.).^19^ *Job control* was assessed with six items (*α* = .87) ranging from 1 = *very little/not at all* to 5 = *very much* (eg 'Can you organize your workday independently?').^18^

#### 2.2.3 Health and productivity outcomes

*Self‐rated health* was measured with one item ranging from 1 = *very bad* to 5 = *very good* ('How would you describe your state of health in general?'). *Psychosomatic complaints* were measured with seven items (*α* = .75) ranging from 1 = *never* to 5 = *constantly* (eg 'How often have you suffered from neck or shoulder pain over the last 12 months').^23^ *Exhaustion* was assessed with eight items (*α* = .80) ranging from 1 = *totally disagree* to 4 = *totally agree* (eg 'After my work, I usually feel worn out and weary'.).^24^ *Sick leave* was assessed with one item ('How often did you have to stay away from work due to sickness during the last 6 months (excluding accidents, sports injuries)?').^18^ *Work engagement* was assessed with nine items (*α* = .95) ranging from 0 = *never* to 6 *always/every day* (eg 'At my work, I feel bursting with energy'.).^25^ *Commitment* was assessed with four items (*α* = .84) ranging from 1 = *not true at all* to 7 = *almost fully true* (eg 'I would be very happy to spend the rest of my career with this organization'.).^26^ *Self‐rated productivity* was measured with one item ranging from 0 = *Worst performance* to 10 = *Top performance* ('How would you rate your overall job performance on the days you worked during the past four weeks (28 days)?').^27^ From the separate health questionnaire, the presence of any one *health disorder* from a broad list of 25 was assessed (yes/no), ranging from back pain to cancer ('Did you have any complaint or disorder in the last three months?') (Swiss Health Survey). Furthermore, from a list of various medical substances, *consumption of pain killers* was assessed ranging from 1 = *never* to 5 = *several times daily*, which was dichotomized to yes/no for analysis due to prevalence rates. Similarly, from a list of visits to various medical services, *visits to general physician* (*GP*) and *visits to physiotherapists* were assessed (yes/no).

#### 2.2.4 Demographics

Age, gender, educational level, job position and job tenure were assessed as covariates. Job tenure was logarithmically transformed due to skewness.

### 2.3 Analysis

#### 2.3.1 Computation of the ratio

Two mean factors of job demands and resources were computed from the measures listed above^8^, ^9^ After transforming all scales to a range from 1 to 5, the resources factor was divided by the demands factor, leading to a continuous ratio scale potentially ranging from 0.2 to 5. Finally, the continuous ratio scale was transformed into an ordinal scale with four groups.

#### 2.3.2 Application of markers

These groups serve as "markers" for analysis and subsequent change processes. In line with the ERI ratio,^1^ resources are overwhelmed by demands in the “critical” category (ratio < 1). In reference to the positivity ratio,^17^ resources clearly dominate demands in the “very good” category (ratio ≥ 2). Between these extremes, two intermediate categories were calculated equally. The categories and respective value ranges are displayed in Table 1. The smaller N in comparison to the overall sample results from the possibility of a 'no answer' category in one of the resources scales, which has an impact on the overall ratio in terms of missing values.

**Table 1:** Value range and frequencies of the ratio groups

Label	Value range	JSA N (%)	HQ N (%)
Critical	0.200‐0.999	304 (11.1%)	51 (10.2%)
Moderate	1.000‐1.499	1336 (48.8%)	267 (53.5%)
Good	1.500‐1.999	787 (28.7%)	143 (28.7%)
Very good	2.000‐5.000	311 (11.4%)	38 (7.6%)
Total		2738 (100%)	499 (100%)

Abbreviations: HQ, health questionnaire; JSA, job stress analysis.

#### 2.3.3 Statistical analysis

We pursued a twofold analysis strategy: First, we conducted general linear models with the ratio in its ordinal‐scaled and continuous form as a predictor of interval‐scaled health and productivity outcomes from same‐source data (base questionnaire). Sick leave values were corrected for extreme values. We also performed logistic regression analyses with the ratio in its ordinal‐scaled form as a predictor of health outcomes from separate‐source data (health questionnaire). Second, we performed logistic regression analyses with the ratio in its ordinal‐scaled form as a predictor of benchmarked health outcomes.

#### 2.3.4 Benchmarks

Z‐scores were computed in regard to external benchmarks ((X–M‐Benchmark)/SD‐Benchmark) and then dichotomized using the 60% percentile as a cut‐off point. The call for benchmarks comes from companies and practitioners, who work with threshold values as call‐for‐action with a so‐called “traffic light” system: *Green* = no action required; *Yellow* = further monitoring and preventive action recommended (60th percentile); and *Red* = immediate action required (90th percentile). The benchmarks were defined by above mentioned stress researchers and consultants, based on a large sample of the working population in Switzerland.

#### 2.3.5 Comparison of ratio to single factors

We compared variance explained in the outcomes by either using job demands and job resources factors separately (including their interaction term) or by using the ratio (*Hypothesis* 2), conducting ordinary least square regression analyses. The traditionally used goodness of fit index *R* ^2^ is not an adequate indicator for our purpose, as *R* ^2^ is (1) sensitive to the number of predictors^28^ and (2) is not adequate for comparing non‐nested models. However, as we are especially interested in the amount of explained variance, we considered the adjusted *R* ^2^, as it controls for the number of predictors. All analyses were conducted using the statistical software IBM SPSS Statistics 24.

## 3 RESULTS

All analyses consistently showed that a better ratio of resources to demands was associated with a decrease in health problems and an increase in productivity outcomes (see Table 2). This confirms Hypothesis 1 for the same‐source data. Similar patterns can be seen for the outcomes of the separate health questionnaire (Table 3), although significance levels are not reached for all measures. Thus, for separate‐source data, Hypothesis 1 can only partially be confirmed. Comparison of the adjusted *R* ^2^ between single predictors (including interaction term) and the ratio reveal a loss in variance explanation up to 4 percentage points when condensing the factors to a ratio, which concerns mainly work engagement and commitment (Table 4). For the other outcomes, the loss in variance explanation ranges from 0.1 to 2.6 percentage points. We consider this as largely comparable, which confirms Hypothesis 2.

**Table 2:** Predicted health and productivity outcomes from same source data (JSA): Results from general linear models using the continuous dependent variable (B) and logistic regression analyses using a dichotomized version based on external benchmarks (OR)

	Self‐rated health (very bad‐very good)				Psychosomatic complaints (never‐constantly)				Exhaustion (low‐high)				Sick leave^i^
	B	OR^a^	CI−	CI+	B	OR^b^	CI−	CI+	B	OR^b^	CI−	CI+	B
Constant	3.95***	4.17***			2.23***	0.49**			1.98***	0.31***			5.30***
Age	0.00	1.00	0.99	1.01	−0.01*, a	0.98**	0.98	0.99	−0.01***	0.98***	0.97	0.99	−0.02
Gender^e^	0.06*, a	1.27*, a	1.03	1.57	0.24***	1.99***	1.66	2.35	−0.01	0.88	0.72	1.05	0.15
Educational level	0.09***	1.36***	1.24	1.49	−0.07***	0.84***	0.77	0.90	−0.01	0.92	0.86	1.01	−0.72***
Job position^f^	0.07*, a	1.22	0.97	1.55	−0.08**	0.88	0.72	1.06	−0.02	0.95	0.78	1.18	−0.34
Job tenure^g^	−0.04**	0.90*, a	0.82	0.99	0.03*, a	1.11**	1.03	1.20	0.03**	1.05	0.98	1.15	0.01
R/D‐Ratio groups^h^													
Critical	−0.64***	0.15***	0.09	0.25	0.74***	8.53***	5.86	12.43	0.87***	19.42***	12.47	30.23	1.91***
Moderate	−0.34***	0.29***	0.17	0.45	0.37***	2.95***	2.18	4.00	0.48***	4.93***	3.36	7.23	1.00**
Good	−0.16**	0.57*, a	0.36	0.90	0.13**	1.64**	1.19	2.26	0.20***	1.50†, b	1.00	2.27	0.38
Very good^h^	—	1.00	—	—	—	1.00	—	—	—	1.00	—	—	—
Adjusted *R* ^2^/Nagelkerke	0.08	0.09			0.15	0.14			0.23	0.20			0.03
R/D‐Ratio cont.	0.37***	3.73***	2.84	4.89	−0.45***	0.24***	0.19	0.29	−0.54***	0.09***	0.07	0.12	−1.06***
Adjusted *R* ^2^/Nagelkerke	0.08	0.09			0.15	0.15			0.23	0.20			0.03
N	2679				2676				2678				2648

	Work Engagement^d^ (never‐always)				Commitment (low‐high)				Self‐rated productivity^i^ (0%‐100%)
	B	OR^c^	CI−	CI+	B	OR^c^	CI−	CI+	B
Constant	3.34***	1.14			4.12***	1.00			75.53***
Age	0.02***	1.03***	1.02	1.04	0.03***	1.04**	1.04	1.06	0.18***
Gender^e^	0.21***	1.27*, a	1.03	1.57	−0.21***	0.74**	0.61	0.88	0.11
Educational level	0.01	1.13*, a	1.03	1.24	0.01	1.02	0.94	1.11	−0.62*, a
Job position^f^	0.48***	2.46***	1.94	3.13	0.37***	1.88***	1.51	2.34	0.25
Job tenure^g^	−0.10***	0.85***	0.77	0.93	−0.01	0.96	0.89	1.04	0.25
R/D‐Ratio^h^									
critical	−1.16***	0.17***	0.11	0.23	−1.34***	0.14***	0.10	0.21	−9.26***
moderate	−0.55***	0.46***	0.32	0.65	−0.50***	0.40***	0.29	0.55	−5.02***
good	−0.29**	0.71†, b	0.49	1.02	−0.27**	0.65*, a	0.46	0.92	−1.79†, b
very good^h^	.	1.00	.	.	.	1.00	.	.	.
Adjusted R^2^/ Nagelkerke	0.12	0.12			0.18	0.16			0.06
R/D‐Ratio cont.	0.66***	3.10***	2.93	4.02	0.70***	3.51	2.79	4.42	6.17***
Adjusted R^2^/ Nagelkerke	0.12	0.12			0.16	0.16			0.06
N	1977^4^				2678				2658

Abbreviation: CI, confidence interval.

a Odds ratio for experiencing good self‐rated health in relation to external benchmark.

b Odds ratio for experiencing psychosomatic complaints and exhaustion in relation to external benchmark.

c Odds ratio for experiencing high work engagement and commitment in relation to external benchmark

d Reduced sample size as part of voluntary in‐depth scale of JSA

e 0 = men, 1 = woman (0 = reference category).

f 0 = no leadership position, 1 = leadership position (0 = reference category).

g Logarithmically transformed.

h Reference category = +++.

i No benchmarks available.

*** *P* < .001.

** *P* < .01.

* *P* < .05.

^†^ *P* < .10.

**Table 3:** Predicted health outcomes from separate source data (health questionnaire): Results of logistic regression analyses

	Disorders (no‐yes)			Consumption pain killers (no‐yes)			Visits to general practicioner (no‐yes)			Visits to physiotherapist (no‐yes)		
	OR*, a	CI−	CI+	OR*, a	CI−	CI+	OR*, a	CI−	CI+	OR*, a	CI−	CI+
Constant	0.50			0.36			0.30			0.08		
Age	1.01	0.99	1.04	1.00	0.98	1.03	1.03*	1.00	1.05	1.04*	1.01	1.08
Gender†, b	2.88***	1.71	4.87	2.10**	1.38	3.20	0.91	0.59	1.40	2.12*	1.11	4.06
Educational level	1.09	0.87	1.36	0.90	0.74	1.10	1.03	0.83	1.26	0.70*	0.52	0.96
Job position^c^	0.74	0.45	1.20	0.90	0.57	1.42	0.44**	0.27	0.72	1.27	0.61	2.64
Job tenure^d^	1.01	0.83	1.23	0.98	0.83	1.16	1.04	0.87	1.24	0.86	0.67	1.11
R/D‐Ratio^e^												
Critical	3.85*	1.21	12.32	4.96*	1.76	13.98	1.26	0.49	3.23	3.14	0.71	13.97
Moderate	1.83	0.81	4.13	2.22†	0.94	5.22	0.49†	0.22	1.08	1.81	0.47	6.96
Good	1.26	0.54	2.90	2.53*	1.05	6.10	0.71	0.31	1.59	1.30	0.32	5.26
Very good^f^	1.00	—	—	1.00	—	—	1.00	—	—	1.00	—	—
Nagelkerke *R* ^2^	0.11			0.09			0.09			0.09		
R/D‐Ratio cont	0.43**	0.24	0.76	0.58*	0.34	0.98	1.10	0.65	1.89	0.40*	0.17	0.96
Nagelkerke *R* ^2^	0.11			0.07			0.06			0.09		
N	424			449			449			446		

Abbreviation: CI, confidence interval.

a Odds ratio for dichotomized outcome (0 = no/ 1 = yes).

b 0 = men, 1 = woman (0 = reference category).

c 0 = no leadership position, 1 = leadership position (0 = reference category).

d Logarithmically transformed.

e Reference category = +++.

*** *P* < .001.

** *P* < .01.

* *P* < .05.

^†^ *P* < .10.

**Table 4:** Comparison of explained variance (ratio vs separate predictors): Results from ordinary least square regression analyses and logistic regression analyses

	Predictors^a^	Ratio^b^	
	Adjusted *R* ^2^/Nagelkerke	Adjusted *R* ^2^/Nagelkerke	Delta
Self‐rated health	0.091	0.077	0.014
Psychosomatic complaints	0.164	0.147	0.017
Exhaustion	0.258	0.232	0.026
Sick leave	0.031	0.029	0.002
Work engagement	0.157	0.119	0.038
Commitment	0.205	0.163	0.042
Self‐rated productivity	0.070	0.059	0.011
Disorders^c^	0.120	0.108	0.012
Consumption pain killers^c^	0.072	0.068	0.004
Visits to general practicioner^c^	0.077	0.059	0.018
Visits to physiotherapist^c^	0.102	0.089	0.013

a Factors of job resources, job demands and their mean‐centered interaction term.

b Ratio as continuous predictor.

c Dichotomous variable.

## 4 DISCUSSION

The aim of this study was to show how a ratio of job resources and demands could be translated into the language of company stakeholders and consultants while retaining scientific value for researchers in the field of work, stress and health.

### 4.1 Better health and productivity with each step of the ratio

The findings from same‐source data showed clear patterns of increase in a variety of health and productivity measures with each step of increase in the ratio. The most pronounced odds ratios and B values were found for exhaustion: The group with a critical ratio is nearly twenty times more likely to feel worn out at work and after work. This may also reflect its work‐related operationalization, compared to self‐rated health and psychosomatic complaints that are assessed in general. Looking at self‐rated productivity, the critical group faces a nine percentage points reduction in work performance (the scale is handled as a 0%‐100% performance scale) and misses work due to sickness four days a year more often than the very good ratio group. Findings from separate‐source data were more ambiguous: The group with the critical ratio has four times higher odds for prevalence of any disorder and five times higher odds of consuming pain killers. Visits to GPs seem to be U‐shaped, whereas visits to physiotherapist decrease more or less linearly with improvement of the ratio. However, these results are non‐significant, which is partly due to the small numbers of participants in the latter groups (see Table 1) and low prevalence in the outcomes of interest, such as visits to physiotherapists.

### 4.2 Predictive power of the ratio

As to be expected, the explained variance differs with the outcome measured: Loss in explained variance due to aggregation of two factors into a single ratio is small for measures which are known to be predicted by both factors simultaneously and, not surprisingly, higher for positive outcomes such as engagement and commitment which are known to be predicted primarily by job resources. A study in the health care sector showed that job resources and demands may explain around 30%‐39% of the variance in emotional exhaustion, which corresponds to the results of this study.^29^ Furthermore, the results lie within the range of the expectable amount of variance that can be explained as the effect of working conditions on general health outcomes.^30^, ^31^

### 4.3 Communication of the ratio

**Figure 1:**
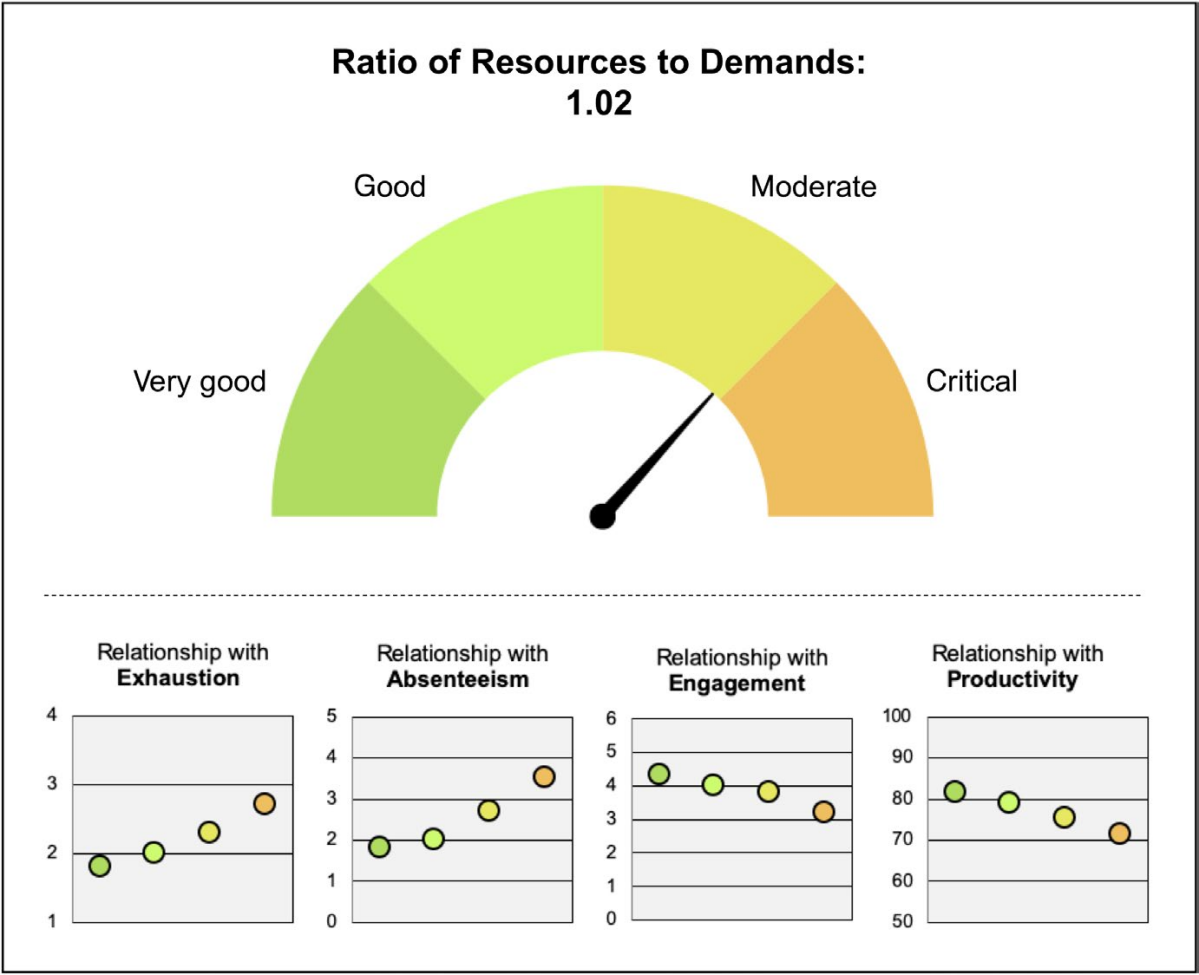
Resources‐Demands Ratio

In regard to the ratio's practical use in companies, we showed a way of translating the ratio of job resources to demands so that it can easily be understood and utilized by company stakeholders and consultants. In order to accomplish this, on the basis of the above results, we present a visualization for company stakeholders (Figure 1). We refer to the ratio as the “Resources‐Demands Ratio” and show both the continuous value of the ratio and the four ratio groups in one graph, marked as “critical” to “very good”. Further, color coding is applied to mark a predominance of resources (green) versus demands (orange). In subjacent graphs, relations of the four groups (again consistently colored) with positive and negative health and productivity outcomes are shown in order to underline the relevance of the ratio in terms of work and health‐related outcomes.

## 5 LIMITATIONS

This study is based on cross‐sectional data, limiting causal statements on the relation between work, health, and productivity, which is known to be multicausal and reciprocal.^30^, ^31^ As many variables were measured with the same instrument, we assessed the effect of an unmeasured latent factor, which accounted for a very small proportion of variance (1.2%), suggesting that common‐method variance was not a predominant problem.^32^ Both data sources were generated by self‐reports. Subsequent studies may apply multi‐method approaches^30^ or shift the level of analysis to work units, to understand how job demands and resources develop dynamically in teams and translate to objectively measured absenteeism rates and productivity measures in these units. From a practical perspective, systematic evidence on the application of such a ratio has yet to be generated: Most companies already gather data on the work situation and are reluctant to adapt their surveys and reporting routines. Further, we do not recommend to use a ratio as a stand‐alone indicator, but as an entry point that triggers discourse and builds a shared mental model, as outlined above. Hereby, when communicating statements such as “a critical ratio is associated with 4 days more sick leave a year”, it must be clear that such evidence is based on cross‐sectional data and constitutes a claim for the average working population. This also requires awareness that specific job demands and resources may interact in differential ways in different contexts, going as far that under certain circumstances the generally positive impact of a resource like social support could cause negative side‐effects. The use of a ratio must therefore be accompanied by awareness that health, well‐being and productivity develops in interaction of a person with different social systems (family, community, company, etc) and organizational levels (individual, groups, leaders, organisation, etc).

## 6 CONCLUSIONS

While the JD‐R model has found wide recognition among researchers, it offers considerable possibilities for organizations. We recommend to use the ratio as a starting point of discourse and action in intervention projects. Based on the ratio, discussions may shift to the values and pattern of the general job demands and job resources factors: For example, are job resources strong enough in view of medium to high demands? Are both resources and demands low, which won't impair health but neither engage workers? Further elaboration will then shift to single scales such as control or work load, and finally to a qualitative, contextualised discussion about specific root factors on individual, group, leader or organizational level.^33^ As stated in the introduction, during these discussions the ratio can serve as a shared mental model to both OHS and HRM experts, and facilitate the vertical alignment of intervention aims such as health and well‐being with organizational goals such as productivity and costs.^34^ Finally, the ratio might also shift focus from (negative) demands to (positive) resources, which offers an alternative perspective to monitoring sickness and abseenteism rates.

## DISCLOSURE

*Approval of the research protocol*: N/A. *Informed consent*: N/A. *Registry and the registration no. of the study/trial*: N/A. *Animal studies*: N/A. *Conflict of interest*: The authors declare no conflict of interests for this article.

## AUTHOR CONTRIBUTIONS

GJJ, GFB and RB conceived the design; GJJ and RB collected the data; GJJ, DF and RB analysed the data; and GJJ, RB and SB led the writing.
